# Arthroscopic iliopsoas tenotomy after total hip arthroplasty: safe method for the right patient

**DOI:** 10.1186/s40634-023-00568-1

**Published:** 2023-01-18

**Authors:** Sarantos Nikou, Ida Lindman, Arnar Sigurdsson, Louise Karlsson, Axel Öhlin, Eric Hamrin Senorski, Mikael Sansone

**Affiliations:** 1 Department of Orthopaedics, Institute of Clinical Sciences, Sahlgrenska Academy, University of Gothenburg, Gothenburg, Sweden; 2 Department of Orthopaedic Surgery, South Älvsborg Hospital, 501 82 Borås, Sweden; 3 Department of Health and Rehabilitation, Institute of Neuroscience and Physiology, Sahlgrenska Academy, University of Gothenburg, Gothenburg, Sweden

**Keywords:** Hip, Iliopsoas impingement, Tenotomy, Total hip arthroplasty, Hip arthroscopy

## Abstract

**Purpose:**

To evaluate the outcome of arthroscopic treatment for iliopsoas impingement after total hip arthroplasty (THA) 2 years after surgery using patient reported outcomes (PROM).

**Methods:**

In this study 12 patients (13 hips) were included from a local hip arthroscopy registry. Patients completed web-based PROMs preoperatively and at a minimum of 2 years postoperatively. The PROMs included the International Hip Outcome Tool short version (iHOT-12), the Copenhagen Hip and Groin Outcome Score (HAGOS), the European Quality of Life-5 Dimensions Questionnaire (EQ-5D), the Hip Sports Activity Scale (HSAS) for physical activity level, the Visual Analog Scale (VAS) for overall hip function and a single question regarding overall satisfaction with the surgery.

**Results:**

The mean age was 64.4 years (±15.1SD), mean body mass index (BMI) was 26.6 (±4.3SD), mean follow-up time was 49.8 months (±25SD). Comparing PROMs preoperatively with 2-year follow up showed an improvement for many of the PROMs used. The PROMs scores were iHOT-12 (24.9 vs 34.5, *p* = 0.13), HAGOS subscales (symptoms 38.2 vs 54.5, *p* = 0.05; pain 36 vs 53, *p* = 0.04; sport 14.1 vs 35.1, *p* = 0.03; daily activity 31 vs 47.5, p = 0.04; physical activity 21.8 vs 24, *p* = 0.76; quality of life 24 vs 35, *p* = 0.03), EQ-VAS (57.9 vs 58, *p* = 0.08), EQ-5D (0.34 vs 0.13, *p* = 0.07) and VAS for overall hip function (43.1 vs 46.2, *p* = 0.14). In total, 10 out of the 12 patients (83%) were satisfied with the intervention.

**Conclusion:**

Patients undergoing surgery for iliopsoas impingement after previous THA showed improved self-reported hip function where most patients were satisfied with treatment.

## Introduction

Total hip arthroplasty (THA) is the gold standard treatment for osteoarthritis (OA) of the hip, with around 15,000 surgeries performed every year in Sweden [38]. Results after THA are generally good in terms of patient satisfaction and hip function, however, persistent pain after THA is not uncommon [16]. In the Swedish hip arthroplasty register (SHAR), with a national coverage of 100% and completeness in primary THA of 96–98%, has 9% of patients reported considerable pain with their operated hip in 2019 [38]. Studies from other countries report that the rate of groin pain after THA ranges from 1 to 18% [2]. It is hence important to study possible causes of pain for these patients as well as possible treatment strategies.

It has previously been suggested that iliopsoas impingement (IPI) is an underdiagnosed adverse event in patients with groin pain after THA, accounting for around 4% of all underlying reasons for groin pain after THA [1, 4, 8]. Patients with iliopsoas impingement often present with groin pain that increases on exertion, as in pain when walking upstairs or raising oneself from a seated position. Clinical signs include pain with resisted straight leg raise and pain in early flexion of the hip [1, 17, 26]. A diagnosis can be made based on symptoms, clinical examination and with the help of radiographic imaging. Ultrasonographic guided injections in the iliopsoas bursa can contribute to the diagnosis of iliopsoas impingement [24]. A malpositioned acetabular component with an excessive anterior cup overhang can be the underlying cause of the iliopsoas impingement [21]. Plain radiographs of the hip and pelvis can be helpful to control the cup placement and size and to exclude signs of loosening of the prosthesis components.

There are several different treatment options that have been explored for management of IPI after THA. These include iliopsoas sheath injections, revision surgery, as well as iliopsoas tenotomy [6, 9, 21]. First line of treatment generally includes physical therapy and corticosteroid injections, with a suggested non-operative treatment regime for at least 6 months [19]. It has previously been documented that these methods are successful in approximately 39–50% of IPI cases [4, 30, 31].

Arthroscopic tenotomy of the iliopsoas tendon is a minimally invasive method previously reported with improved outcomes and low complication rates when compared to other surgical alternatives [6, 21, 25, 31, 32]. Previously, studies have shown up to 92% improved pain scores in patients with IPI after arthroscopic psoas release with less than a 4% complication rate [15].

However, there is a lack of studies reporting on the surgical results using modern validated PROMs commonly used for patients who undergo hip arthroscopy. The aim of this study was to evaluate patient reported outcome measurements (PROMs) and possible adverse effects of arthroscopic iliopsoas tenotomies in a group of patients with IPI after THA.

## Methods

All patients who underwent hip arthroscopy due to suspected IPI following THA in Gothenburg, between January 2016 and December 2019, and who were included in the local hip arthroscopy registry were included in the study. The surgeries were performed at two hospitals by three experienced high-volume orthopedic surgeons. The local hip arthroscopy registry includes all patients who undergo hip arthroscopy in Gothenburg. Patients complete a web-based questionnaire including PROMs preoperatively, and at 2, 5 and 10 years after surgery. Perioperative data such as cartilage lesions, pathological lesions of acetabulum or caput femoris as well as labrum, iliopsoas tendon and ligamentum teres lesions are also registered by the surgeons. Demographic data comprised age, sex, BMI, affected side, symptoms and pain free interval between symptoms onset and THA are reported in -Table 1-. The PROMs used are:

- EQ-5D and EQ VAS (European Quality of Life–5 Dimensions Questionnaire and European Quality of Life–Visual Analog Scale) a standardized instrument evaluating health-related quality of life [28]. It is a descriptive system which comprises five dimensions: mobility, self-care, usual activities, pain/discomfort, and anxiety/depression. Each dimension has 5 levels: no problems, slight problems, moderate problems, severe problems, and extreme problems. Several studies have shown that this instrument is valid and reliable [20, 27].
- HAGOS (Copenhagen Hip and Groin Outcome Score),with 6 parts including symptoms, pain, function in daily living, function in sports and recreation, participation in physical activities, and hip- and/ or groin-related quality of life [34]. HAGOS is validated in Swedish and is a reliable and responsive instrument [33].
- HSAS (Hip Sports Activity Scale),an instrument measuring the level of physical activity [23].
- A VAS scale for hip function.
- A single question regarding patient satisfaction (yes/no).
- iHOT-12 (International Hip Outcome Tool short version), a shorter version of the iHOT-33 which measures both health-related quality of life and changes after treatment in young, active patients with hip disorders [14].

**Table 1:** Patient demographics

Total number of patients	12
Total amount of hips	13
Age-mean, years (SD)	64.4 (±15.1)
Male/Female (%)	4/8 (33.3/66.7)
BMI^a^ (SD)	26.6 (±4.33)
Operated side, right/left (%)	5/8 (38/62)
Time since prosthesis-median, months (min-max)	42 (21–100)

^a^*BMI* body mass index

If patients had failed to respond to the 2-year postoperative questionnaire, they were contacted again by phone and a new questionnaire was sent out to them, regardless of the amount of time passed since surgery.

**Fig. 1:**
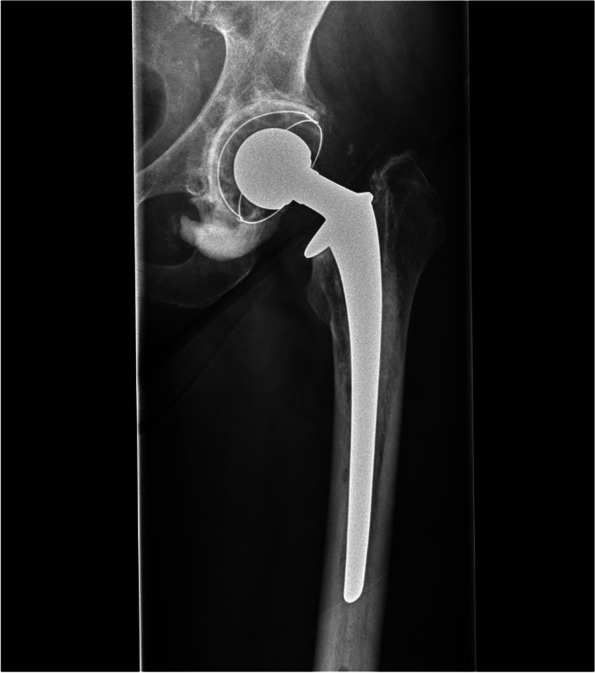
Total hip arthroplasty, anteroposterior radiograph. Postoperative radiograph after a total hip arthroplasty. Notice the cement extrusion at the acetabular side that caused the mechanical irritation of the psoas tendon

**Fig. 2:**
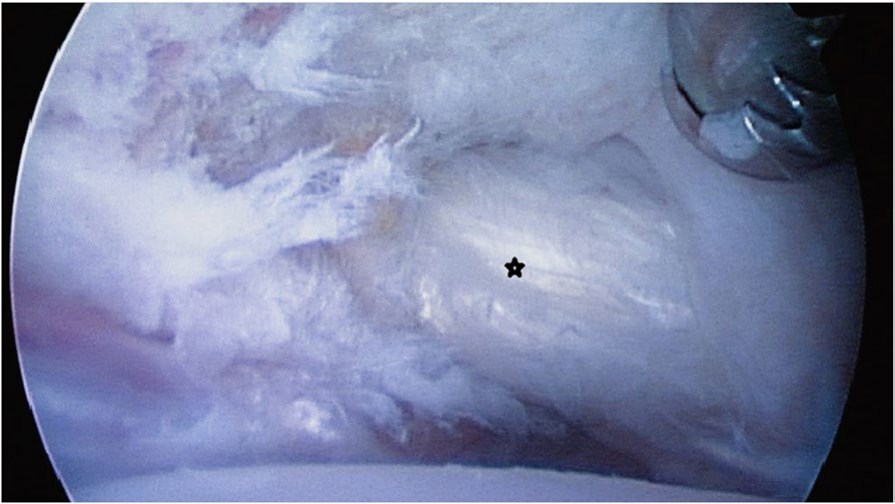
Arthroscopic image of the hip joint. Arthroscopic image in a case of IPI after a total hip arthroplasty. Notice the mildly frayed psoas tendon

**Fig. 3:**
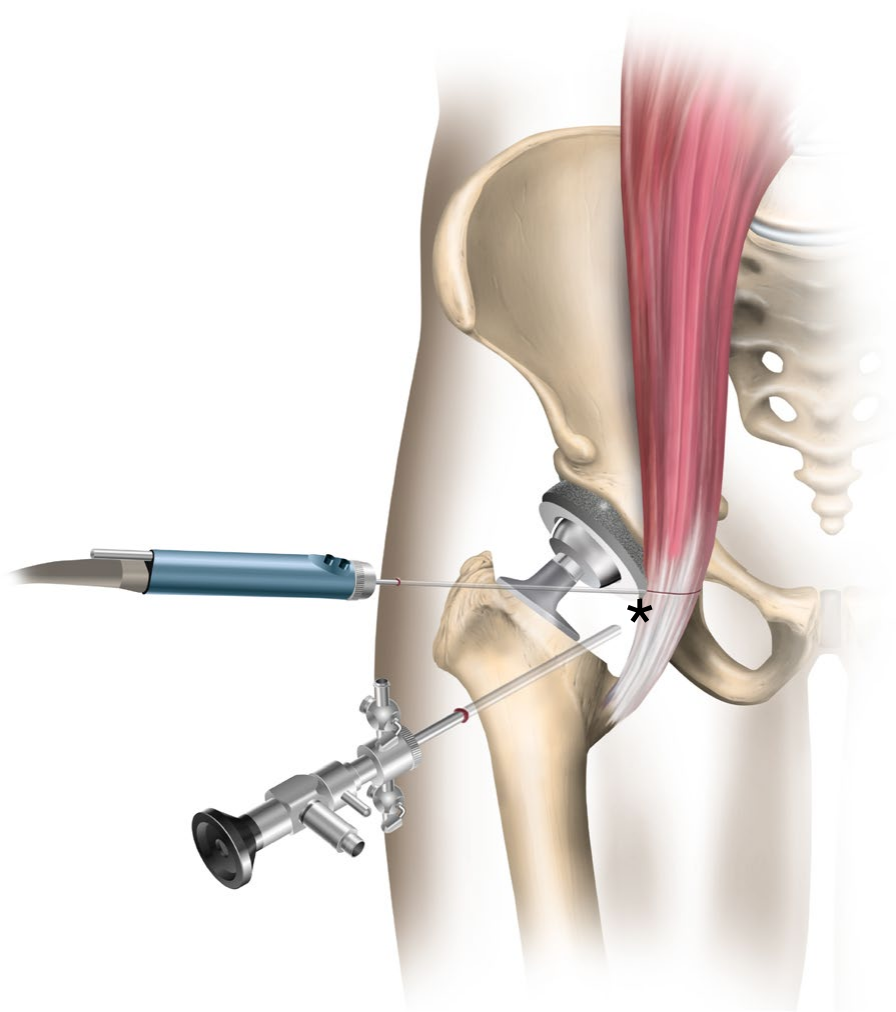
Illustration of an iliopsoas tenotomy. Illustration of iliopsoas impingement because of a large protruding acetabular cup. Tenotomy of the irritated psoas tendon is performed at the level of the cup

Clinical diagnosis was based on patient history, clinical signs and ultrasound guided diagnostic block with local anaesthetics with or without steroids. A positive diagnostic block was a strong indicator to proceed with surgery. Diagnostic evaluation using x-rays (anteroposterior, lateral and pelvis views) as well as computer tomography (CT) scans were used. Inclination along with protrusion was evaluated by an independent orthopaedic surgeon (Figs. 1, 2, 3).

Surgical procedure was performed in a supine position, using 2 arthroscopic portals, one anterolateral and one mid-anterior. No traction was used, and the procedure was performed with standard pump pressure. The tendon was located at the level of the anteromedial aspect of the acetabular cup and complete tenotomy was performed under direct visualisation.

Postoperatively the patients were allowed to fully weight-bear as tolerated and if needed to use two crunches for the first few days. Physical therapy started immediately without any restrictions. We routinely prescribe nonsteroidal anti-inflammatory drugs (NSAIDs) postoperatively to prevent heterotopic ossification (HO) if not contraindicated.

For evaluation of post-operative complications, the Clavien–Dindo Classification was used [5]. This is a standardized classification system (grade I-V) used in order to report postoperative complications originally used in general surgery and validated for use in orthopaedic surgery [3].

### Statistics

Continuous demographic variables are analyzed with descriptive statistics and presented as mean and standard deviation (SD), and median with range. The statistical analysis of the patient data and PROMs was done using the Statistical Package for the Social Sciences (IBM SPSS statistics, version 28.0.1.1). To compare paired means for continuous PROM data not normally distributed non-parametric statistical testing was used. The Wilcoxon signed rank test was utilized to compare the preoperative and postoperative PROM data. Significance level was set at the value of *p*˂0.05. The number of patients exceeding the minimally important change (MIC) for the six HAGOS subscales and the iHOT-12 was reported. The MIC values for the iHOT-12 9.0, HAGOS pain 9.7, HAGOS symptoms 9.3, HAGOS function 11.8, HAGOS physical activity 13.1, HAGOS sports 10.8 and HAGOS quality of life 8.8 were used as previously reported [18, 33].

## Results

The study comprised of 12 patients, 13 hips (8 female, 4 male), with one patient operated bilaterally, treated between January 2016 and December 2019. Average age was 64.5 years (±15.1SD). Mean BMI was 26.6 kg/m^2^ (±4.33SD). Mean onset of symptoms after THA was 9.3 months (±5.5SD, range: 0–52). In 8 out of 13 cases there was no pain-free interval after THA while 5 patients experienced a mean pain-free interval of 24 months (±19.3). A total of 11 out of 13 implants were primary cases while 2 were revisions, 7 out of 13 cases were cemented THA, 4 were THA without cement and 2 were hybrid THA. Out of 13 THA 7 of them were performed with a modified anterolateral approach and 6 cases with a posterior approach. One case underwent later a revision of the cup component because of aseptic loosening.

Pre-operative and postoperative PROM scores are presented in Table 2.

**Table 2:** Patient Reported Outcome Scores Preoperatively and at Minimum 2-year Follow Up

Outcome	Preoperative, mean	2y-follow up postoperative, mean	Change	*p* value
iHOT-12^a^	24.9 ± 13.8	39.5 ± 19.6	15.6 ± 22.1	0.13
HAGOS^b^ -symptoms	38.2 ± 17.6	54.5 ± 33.1	23.9 ± 33	0.05
HAGOS-pain	36 ± 18.3	53 ± 30.3	24.5 ± 27.5	0.03
HAGOS-sport	14.1 ± 10.4	35.1 ± 22.1	24.4 ± 27.4	0.03
HAGOS-daily activity	31 ± 23.5	47.5 ± 28.6	22.5 ± 30	0.04
HAGOS-physical activity	21.8 ± 22.5	24 ± 21.9	2.5 ± 21.1	0.76
HAGOS- quality of life	24 ± 10.7	35 ± 20.9	14.5 ± 16.2	0.03
EQ 5D^c^	0.339 ± 0.368	0.127 ± 0.385	−0.608 ± 0.076	0.07
EQ-VAS^d^	57.9 ± 15.9	58 ± 22.4	9.9 ± 14.3	0.08
VAS-Hip function	43.1 ± 17.9	46.2 ± 14.8	8.6 ± 13.8	0.14
Satisfied		10 (83%)		
Not satisfied		2 (17%)		

^a^iHOT-12 International Hip Outcome Tool

^b^HAGOS Copenhagen Hip and Groin Outcome Score

^c^EQ-5D EuroQoL-5 Dimension Questionnaire

^d^VAS visual analogue scale

Out of 12 patients 10 (83%) reported that they were satisfied with the surgery.

In 8 of the patients there were signs of irritation, such as thickening, redness and vascularization of the psoas tendon during arthroscopy as reported by the treating surgeon. In 4 out of 13 hips there was a clinically visible protrusion of cup or cement visibly irritating the tendon.

Of the six HAGOS subscales 54% of patients exceeded the MIC for HAGOS pain, 46% for HAGOS symptoms, 38% for HAGOS function, 38% for HAGOS physical activity, 46% for HAGOS sports, 54% for HAGOS quality of life. 31% exceeded the MIC for the iHOT-12.

Table 3 shows complementary examinations. Radiological results found a mean frontal inclination of 44.6 ° (range: 31–52°; 95%CI), and anterior projection on oblique view 8.71 ° (range − 1-14, 95%CI). Computed tomography (CT) found mean anteversion of 18.7 ° (range: 11–37; 95%CI).

**Table 3:** Radiographic findings of cup placement on plain x-rays and CT^a^

	Mean	Range
Frontal inclination x-rays	44.6 ± 6.9	31–51 (95%CI)
Anterior projection x-rays	8.71 ± 4.5	1–14 (95%CI)
CT anteversion	18.7 ± 6.8	11–37 (95%CI)

^a^CT Computed tomography

For the group of 5 patients with mean pain-free interval of 24 months the PROMs were iHOT-12 (35.9 vs 42.4, *p* = 0.29), HAGOS subscales (symptoms 48.2 vs 63.4, *p* = 0.47; pain 48.8 vs 65.6, p = 0.47; sport 19.5 vs 33.6, *p* = 0.27; daily activity 37.5 vs 45, *p* = 0.47; physical activity 18.7 vs 21.9, *p* = 0.85; quality of life 27.5 vs 41.3, *p* = 0.14), EQ-VAS (59.8 vs 66.5, *p* = 0.14), EQ-5D (0.5 vs 0.41, *p* = 0.07) and VAS for overall hip function (51.3 vs 53.3, *p* = 0.66). The radiological results for this group found a mean frontal inclination of 51 ° (range 47 ° -54 °, 95%CI), and mean anterior projection on oblique view 6.8 ° (range − 1 ° -13 °, 95%CI). Mean anteversion on computed tomography 21.3 ° (range 11 ° -33 °, 95%CI) .

None of the patients needed postoperative hospital admission. One patient had a Clavien – Dindo score of one (non-life-threatening complication requiring transient medication and resolves within the next 72 h.) because of hip pain radiating distally that resolved a short time after surgery. Arthroscopy did not reveal metallosis in any of the patients.

## Discussion

The most important finding in this study was that most of patients reported clinical satisfactory results and improvements in PROMs postoperatively, at a minimum of 2 years after undergoing arthroscopic tenotomy for iliopsoas impingement following THA. There was an improvement in VAS hip function and most of the HAGOS subscales. Furthermore, the rate of adverse effects was low and similar to previous studies [12, 21].

These findings are in concordance with other studies showing improvement in various PROMs after arthroscopic tenotomy for iliopsoas impingement secondary to THA [8, 15, 37]. Viamont-Guerra et.al [37] reported results on 48 patients treated with arthroscopic tenotomy and found statistical significant improvement postoperatively of both the modified Harris Hip Score (mHHS) and Harris Hip Score (HHS). Tassinari et.al [32] in a series of 16 patients showed improvement in WOMAC score (Western Ontario and McMaster Universities Arthritis Index) at a mean follow up of 27(±20.1SD) months. Guicherd et.al [15] in a prospective series with 64 patients reported improvement of OHS (Oxford Hip Score), patient satisfaction and anterior hip pain at a mean follow-up of 8 months. Di Benedetto et.al [8] reported a series of 13 patients with significant improvement HHS and Medical Research Council (MRC) scale at a mean follow-up of 10 months .

In this study 10 out of 12 (83%) patients were satisfied with the surgery. This is in concordance with a previous study of a group of 16 patients treated with iliopsoas tenotomy after THA that had a satisfaction rate of 87% (16). Similar outcomes have been reported in other smaller studies such as the study by Van Riet A et al. [36] with a series of 9 patients and Gédouin et al. [13] with a series of 10 patients.

It has previously been suggested that careful selection of operative candidates for arthroscopic release of iliopsoas tendon is important for the success of the operation [6]. It has been shown that patients with cup prominence of less than 8 mm have a better chance of successful groin pain resolution with non-operative treatment than patients with cup prominence over 8 mm [4]. In this study it was noted that mean protrusion was 8,7 mm (1–14 mm) which is in concordance with other studies on the subject [14, 15, 17, 18].

The position of the cup after THA correlates with the risk of postoperative iliopsoas impingement [35]. Inclination and anteversion of the cup in the present study was found to be 44,6° (31–52) and 18,7° (11–33), respectively, which is in line with previous literature [22, 35]. One patient in our study had an inclination over 50° (52) and this patient later underwent revision surgery.

Iliopsoas tenotomy in the native hip has been reported on and previous studies have warned about the risk of inducing instability [7, 11, 29]. This may also be the case in THA patients after iliopsoas tenotomy. Guicherd et al. [15] had one case of anterior dislocation in their cohort after transcapsular tenotomy. This is the only case reported that authors of this article are aware of. In accordance with this, no cases of postoperative dislocation were seen in this series. The potential loss of stability after iliopsoas tenotomy may be smaller than the inherent stability that the THA gives.

Post-operative complication rates after arthroscopic iliopsoas tenotomies were low in this study as reported by previous studies [17, 18]. One patient reported a Clavien-Dindo score of one because of postoperative hip pain radiating distally that resolved a short time after surgery. Another patient has since undergone a revision THA surgery. This patient may be an outlier since symptoms started 52 months after THA. The interpretation can be that the symptoms were initially caused by the complication attributed to the implant and not due to IPI. Though fluid extravasation is a known complication that can occur during a hip arthroscopy procedure, and especially if an iliopsoas tenotomy is performed, none of the patients in this group had this specific complication [10]. Common complications of fluid extravasation are abdominal compartment syndrome, metabolic acidosis and hypothermia. Precautions that could be taken are the use of transparent drapes, palpating the thigh and the abdomen intraoperatively, calculating the intraoperative fluid deficit and trying to avoid prolonged operative time [39].

In this patient group the iliopsoas release was performed at the level of the acetabular cup. There are some theoretical advantages compared to releasing the tendon at the lever of the lesser trochanter. The muscular portion of the iliopsoas is greater intraarticularly thus preserving a greater part of the function by performing the release at this level. Additionally, the acetabular cup is routinely checked for any obvious signs of loosening and the tendon for signs of inflammation or structural damage [22].

There are several limitations to this study. It is a retrospective analysis of prospectively collected data. The small patient sample imposes certain limitations about the conclusions that can be drawn from this study. A power analysis was not conducted prior to analysis as this was a retrospective study. That means that there is a significant possibility of type-II error. However, to the knowledge of the authors, this is one of the few current studies evaluating results after arthroscopic psoas tenotomy after THA using recommended PROMs for hip arthroscopy patients.

Although a thorough search was executed with both the local registry data as well as a search in the hospital registers there is still a risk that not all patients were found. However, the risk is deemed to be small.

Another limitation of this study is the short follow-up of 2 years. A longer follow-up could be warranted in order to strengthen conclusions about the long-term effects of this method. Another aspect is that the radiological analysis was conducted by only one surgeon without any inter- or intra-observer agreement analysis of the radiological findings, potentially limiting the accuracy of the radiological values registered. In addition, the hip flexion muscle strength pre- and postoperatively was not measured thus not being able to examine the effect of iliopsoas tenotomy on the hip flexion muscle strength.

## Conclusion

In this study, after arthroscopic treatment of iliopsoas impingement in patients who have previously undergone THA it was shown an increase in VAS hip function and most of the HAGOS subscales at minimum 2-year follow up. In this patient group,10 out of the 12 patients (83%) were satisfied with the surgery.

## Data Availability

The datasets used and/or analyzed during the current study are available from the corresponding author on reasonable request.
